# Interleukin-6 as a keystone cytokine in experimental rat models of chemotherapy-induced peripheral neurotoxicity

**DOI:** 10.1038/s41598-025-34830-6

**Published:** 2026-01-13

**Authors:** Olga Tarasiuk, Alessia Chiorazzi, Alberto Argentini, Elisa Ballarini, Annalisa Canta, Valentina Alda Carozzi, Eleonora Pozzi, Paola Alberti, Valentina Fabbro, Federico Iseppon, Maria Foti, Cristina Meregalli

**Affiliations:** 1 School of Medicine and Surgery, University of Milano-Bicocca, via Cadore 48, 20900 Monza, Italy; 2 NeuroMI (Milan Center for Neuroscience), University of Milano-Bicocca, Monza, Italy; 3 Fondazione IRCCS San Gerardo dei Tintori, Monza, Italy

**Keywords:** Chemotherapy, IL-6, GRO/KC(CXCL1), Cytokines, Neuroinflammation, Paclitaxel, Oxaliplatin, Neuroscience, Neurology

## Abstract

Chemotherapy-induced peripheral neurotoxicity (CIPN) is one common side effect associated with antineoplastic agents. Despite its relevance, the underlying mechanisms remain poorly understood. In this study, we explored cytokines and macrophages that contribute to two chronic models of CIPN comparing paclitaxel (PTX)- and oxaliplatin (OHP)-induced CIPN in rats. To detect chemotherapy-induced sensory or neuronal abnormalities behavioral tests, pathological and morphometric analysis were used. To investigate systemic inflammation, a panel including IL-6, IL-1β, IL-2, IL-10, GRO/KC(CXCL1), TNFα, IFNγ were tested in serum. Besides, we analyzed dorsal root ganglia (DRG), caudal nerves and spinal cord mRNA expression of a panel of pro-inflammatory markers IL-6, CCL2 and NLRP3, as well as caudal macrophage infiltration. We evidenced a remarkable difference in caudal macrophage infiltration in PTX- vs. OHP-treated rats, which was associated with serum CXCL1 and to a different IL-6 expression pattern in serum, DRG, spinal cord and caudal nerve. Moreover, CCL2, NLRP3 upregulation was also increased in the caudal nerves in PTX-treated animals. On the contrary, OHP induced IL-6 and CCL2 expression in DRG. Since our results suggest that CXCL1 or IL-6 could be considered as critical lynchpins between inflammation pain and CIPN, targeting early inflammatory events could be a promising therapeutic approach.

**Supplementary Information:**

The online version contains supplementary material available at 10.1038/s41598-025-34830-6.

## Introduction

Chemotherapy-induced peripheral neurotoxicity (CIPN) is a frequent adverse side effect related to various antineoplastic drugs treatment, which often compromise patients’ outcomes and survival rates^1^. In fact, in cases of severe CIPN, adjustments to chemotherapy dosage or treatment discontinuation are the only options to prevent permanent neuronal damage. CIPN manifests mostly as sensory neuropathy, with less frequent and severe motor and autonomic nervous system alterations. The observed symptoms exhibit a spectrum of severity and duration, ranging from negative symptoms (i.e., hypoesthesia) involving both small (e.g., thermal sensation) and large fibers modalities (e.g., proprioception), to positive symptoms such as paresthesia and/or neuropathic pain. The clinical picture can be persistent due to irreversible nerve damage. CIPN arises through multiple, agent-specific mechanisms including mitochondrial dysfunction, axonal degeneration, and altered ion channel expression^2–4^. For example, paclitaxel (PTX), commonly used to treat breast, ovarian, prostate, and lung cancers, is an anti-tubulin agent involved in axonal transport disruption, while oxaliplatin (OHP) used to treat gastrointestinal cancers, is a platinum-derived drug with primary damage of the sensory neurons of dorsal root ganglia (DRG) resulting in secondary axonal damage, induce chronic, distal, symmetrical sensory peripheral neuropathy, often accompanied by persistent neuropathic pain^5^. Notably, PTX and OHP also induce acute painful symptoms either during or soon after infusion^6^. The exact mechanisms causing the neuronal damage induced by anticancer neurotoxic drugs are not fully understood, but inflammation is increasingly recognized as playing a significant role in the pathophysiology of CIPN, involving activation of glial cells, increased pro-inflammatory cytokines, and recruitment of immune cells in the DRG and spinal cord^7–9^. Across taxanes, platinum, vinca alkaloids, and proteasome inhibitors, preclinical and clinical studies report elevation of pro‑inflammatory cytokines (e.g., TNF‑α, IL‑1β, IL‑6), and relative reductions in anti‑inflammatory IL‑10^8,10,11^. These inflammatory responses not only accompany neurodegenerative changes but may also sensitize sensory neurons and exacerbate neuropathic pain.

Despite various studies describing the mechanisms underlying CIPN, a major gap remains in our understanding of how these mechanisms are temporally and spatially activated in an agent-specific manner, and in particular, if the induced inflammatory response is able to lead to a significant increase in the circulating levels of pro-inflammatory cytokines. In particular, the precise timing and localization of drug-dependent neuroinflammatory responses in CIPN are still not fully elucidated. One objective of the current research is to address this gap by building on our previous findings on CIPN, with a focus on characterizing the neuroinflammatory processes that contribute to disease progression. A second key knowledge gap is the limited number of studies that directly compare different chemotherapeutic agents within the same experimental framework. Such comparative studies are crucial to uncover new mechanistic insights into the agent-specific pathogenesis of CIPN. Understanding these distinctions is critical, as it underscores the importance of tailoring therapeutic strategies to the unique pathophysiological features of each chemotherapeutic drug. Recent findings suggest that different agents may induce distinct patterns of neuroinflammatory responses, both anatomically and at the cellular level^8,12,13^. To gain deeper insight into the role of neuroinflammation in CIPN, we have set up 2 models with different damage sites, PTX- and OHP-induced CIPN in rats and we compared their macrophages infiltrations and cytokines signaling at level of the nervous system and serum at different time points.

## Material and methods

### Ethical statement

The care and husbandry of animals complied with the ARRIVE guidelines and were conformed with the institutional guidelines in compliance with national (D.L.vo n. 26/2014) and international laws and policies (European Union directive 2010/63/UE; Guide for the Care and Use of Laboratory Animals, U.S. National Research Council, 1996). The study of pain in animals followed guidelines outlined by the International Association for the Study of Pain (IASP). The study plan and the procedures were approved and authorized by the ethics committee of the University of Milano-Bicocca (authorization number 0041156/21) and by the Italian Ministry of Health (authorization number 449/2020-PR).

### Experimental models

One hundred sixty-eight Wistar male rats (250–275 gr at the beginning of the study) were purchased from Envigo (Udine, Italy) and were randomized into two different experiments. For each experiment, eighty-four rats were randomized in two treatment groups (forty-two rats/group) consisting of twenty-one animals each for two time points: mid-treatment and end of treatment. To ensure group homogeneity, animals were randomized based on behavioral values at baseline. The estimate of the sample size per group has been derived from our previous studies^14^ where thanks to a fundamental prior power calculation based on laboratory reference values^15^, the assessment of peripheral neuropathy and behavioral investigation were strongly demonstrated. A total of 21 animals per group/each time points were included in the behavioral study to achieve sufficient statistical power. From this cohort, a subgroup of 7 animals per group/each time points were randomly selected for the analysis of inflammatory markers. Serum and tissues were collected from these animals, and the onset of neuropathy was confirmed through morphological and morphometric analysis of their DRGs and peripheral nerves. In respect of the “reduction” principle of the 3Rs, the biological samples harvested were employed for morphological and molecular analysis described in this study and for other experimental research performed at the Experimental Neurology Unit of the University of Milano-Bicocca.

In the first experiment, a vehicle (VEH_PTX_) group was intravenously (i.v.) treated once a week, with 10% EtOH 100%, 10% Tween 80, and 80% saline solution, while the treated group was administered i.v. with PTX 10 mg/kg once a week. All animals were administered for two (mid-treatment) or four weeks (end of treatment)^16^. In the second experiment, a vehicle group was treated i.v. with glucose solution 5% (VEH_OHP_) once a week, and the other group was treated i.v. with OHP 5 mg/kg twice a week^17^. All animals were treated for three (mid-treatment) or six weeks (end of treatment). Both chemotherapy-induced peripheral neurotoxicity models were previously characterized in terms of neurophysiological and histopathological aspects^16,17^. The animals were housed 2/3 per cage in a certified and limited-access facility with controlled temperature (21 °C ± 2), humidity (50% ± 20), and an artificial 12:12 h light cycle from 7 a.m. to 7 p.m.. Mortality was evaluated daily, and body weight changes were measured during the treatment period to monitor general conditions and to adjust the dose of administration. Additionally, throughout the study, a certified veterinarian monitored the rats daily for any signs of debilitation caused by the drug treatments to determine if they should be withdrawn. Such signs included changes in appearance (i.e., piloerection, kyphosis, mucosal dehydration, rhinorrhea), behavior (decreased grooming, eating, and drinking), and activity (decreased exploring and nesting). In both experiments, after every time point, the animals were sacrificed for blood and sample collection. In particular, the caudal nerve was used for histopathological and immunohistochemistry analysis, L4-L5 DRG were obtained for morphometric and morphological analyses, and paw tissues were involved in a measure of intraepidermal nerve fibers density. The blood was used to quantify patterns of cytokines and chemokines in rat serum.

### Behavioral assessments

All the behavioral tests were performed at baseline and mid-treatment as well as baseline and at the end of treatment by an experimenter who was blind to the various treatment groups of the animals.

#### Dynamic test

Dynamic Aesthesiometer Test (model 37,450, Ugo Basile Biological Instruments, Comerio, Italy) was used to assess the mechanical withdrawal threshold in PTX- and OHP-treated animals, as previously described^18^. In detail, after 10 min of acclimatization, a 0.5 mm diameter pointed metallic filament was positioned under the plantar surface of both hind paws; the filament exerted a progressive pressure (up to 50 g within 20 s) and three measures for each hind paw were recorded. The mechanical withdrawal threshold is the maximum pressure exerted (in grams) that triggers paw withdrawal.

#### Plantar test

The paw withdrawal threshold in response to an infrared heat stimulus was assessed using a Plantar instrument (Ugo Basile Biological Instruments), following the protocol detailed in Meregalli et al.^19^. The latency of hind paw withdrawal (expressed in seconds) in PTX-treated rats was recorded. Specifically, the measurement for each paw was taken three times and the final result was calculated as the average of four repeated trials.

#### Cold plate test

Changes in cold hyperalgesia following OHP treatment was monitored using the Cold Plate apparatus (Model 35,100—Cold Plate, Ugo Basile Biological Instruments, Comerio, Italy) to assess the cold nociceptive threshold. This instrument comprises a thermostatic plate set at 4 °C during the test, enclosed by a Plexiglass cylinder. Briefly, each animal was placed inside the Plexiglass holding cage, where it was free to move and walk. An operator then recorded the number of pain/suffering behaviors (including hind paw lifts, flicking/licking, and jumping) over a 5-min trial. The trial was immediately suspended if the animal exhibited signs of severe thermal sensitivity (e.g., evident anxiety and vocalization)^20^.

### Peripheral neurotoxicity

#### Morphological and morphometrical analysis of DRG

To verify DRG neuron damage, we performed a morphological and morphometrical analysis of OHP-treated DRG neurons. This study was performed only for OHP-treated samples since for PTX-treated tissues we already demonstrated that PTX did not induce any DRG neurons size alterations^21^. In detail, 1.5 µm-thick sections stained with toluidine blue were observed with a Nexcope Ne920 AUTO light microscope (TiEsseLab, Milano, Italy) at a magnification of 20X and then analyzed with a computer-assisted image analyzer (Image J software, USA National Institutes of Health). Serial sections spaced 25 µm were collected and the somatic, nuclear, and nucleolar size of DRG sensory neurons were measured on at least 200 DRG neurons/rats from 3 rats/group in randomly selected sections spaced more than 50 µm. Only DRG sensory neurons with a nucleolar area larger than 3 µm were evaluated^22^.

#### Pathological examination and morphometrical evaluation of caudal nerves

At every time point, after sacrifice under terminal CO_2_ anesthesia, caudal nerves were isolated and dissected out for morphological and morphometrical analyses. The samples were fixed by immersion in 3% glutaraldehyde in 0.12 M phosphate buffer solution and post fixed in OsO_4_, then epoxy resin embedded. Specimens were cut, and 1.5 μm semithin sections were obtained, stained with toluidine blue for light microscopy and examined for morphological analysis with a Nexcope Ne920 AUTO light microscope (TiEsseLab Srl, Milano, Italy) at a magnification of 40X. To assess the loss of fibers after PTX treatment with morphometry, the images acquired with the same microscope at a 60X magnification were analyzed using a computer-assisted image analyser using the Image Pro-Plus software (Media Cybernetics). The density (fibres/mm^2^) was measured in randomly selected sections according to previously reported methods^23^.

#### Skin biopsy: intraepidermal nerve fibers (IENF) density evaluation

At both time points, the skin of the hind paw footpad was collected, fixed in PLP 2% (paraformaldehyde 8%, L-lysine, and periodate sodium) for 24 h and stored at −20 °C until the analysis. 20 μm-thick sections were obtained and three sections from each footpad were immunostained, using a free-floating protocol, with a rabbit polyclonal anti-protein gene product 9.5 (PGP 9.5; Proteintech, Illinois, USA) in combination with biotinylated anti-rabbit IgG^22^. The IENF density was determined as the number of PGP 9.5-positive fibers that cross the dermal–epidermal junction per epidermal length (mm) by the same blinded examiner.

### Simoa assay

Serum was obtained by centrifugation (4 °C, 1,500 g, 15 min) of blood for the simultaneous identification of serum cytokine level from 6/7 animals/group at mid and the end of treatment. Each treated group had its corresponding control group treated with a vehicle (VEH). Simoa Planar Array Rat Cytokine 7-Plex Panel 1 Array (ready-make standard set determined for inflammation identification) was performed using a multiplex immunoassay designed for the Quanterix SP-X™ Imaging and Analysis system according to the manufacturer’s instructions (Quanterix, Massachusetts, USA), IL-1β, IL-2, IL-6, IL-10, GRO/KC(CXCL1), TNFα, IFNγ in rat serum were simultaneously measured^24^. Lower limit of quantification is set by manufacturer as IFNγ −54.5 pg/mL, IL-1β −6.3 pg/mL, IL-2 −6.0 pg/mL, IL-6 −4.8 pg/mL, IL-10 −6.4 pg/mL, GROα/KC −1.6 pg/mL, TNFα - 24.2 pg/mL.

### Real time RT-PCR

Total RNA was isolated from frozen DRG, caudal nerve, and spinal cord of animals by Direct-zol RNA Miniprep kit (R2052, Euroclone, Pero, Italy), according to the instructions of the manufacturer. RNA was further quantified using Omega microplate reader (BMG Labtech, Ortenberg, Germany). Further, 1 µg of total RNA was transcribed to cDNA using High-Capacity cDNA Reverse Transcription Kit (4,368,814 Thermo Fisher, Massachusetts, USA), according to the instructions of the manufacturer. Reverse transcription was performed using a thermal-cycler (Eppendorf, Hamburg, Germany) using the following heating protocol: 10 min at 25 °C, 45 min at 42 °C and 5 min at 99 °C. cDNA was then stored at -20 °C until further use. qRT-PCRs were performed on a 96-well plate, in duplicate, using TaqMan™ Gene Expression Master Mix (4,369,016, Thermo Fisher, Massachusetts, USA) and specific TaqMan probes for each analyzed gene IL-6 (4,331,182, Code: Rn014103330_m1), NLRP3 (Rn04244620_m1), CCL2 (Rn00580555_m1), and housekeeping gene 18S (4333760 T, Thermo Fisher, Massachusetts, USA). 40 ng of transcribed cDNA was used for each reaction. Fluorescence intensity was assessed using 7500 Real-Time PCR System (Applied Biosystem, Massachusetts, USA). Transcripts were normalized to the expression of Ribosomal Protein 18S mRNAs. Each measurement was duplicated. Relative quantification was performed using the comparative threshold cycle (Ct) method, and results were expressed in arbitrary units (AU). Expression levels were calculated as 2 − ΔCt, whereas fold changes were calculated using the 2 − ΔΔCt equation.

### Immunohistochemistry (IHC)

To investigate macrophage infiltration, proximal and distal caudal nerves were dissected, post-fixed in 10% formalin overnight, and then paraffin embedded with HistoPro200 (HistoLine, Milano, Italy). 3 μm-thick longitudinal sections were cut using a Leica RM2265 microtome (Microsystems GmbH, Wetzlar, Germany). Infiltrating macrophages were identified by performing immunohistochemistry using a mouse anti-CD68 antibody (Biorad MCA341GA, California, USA). Slides were deparaffinized with xylene and hydrated in descending grades of ethanol. To preserve caudal nerve tissue integrity, antigen retrieval was not performed on nerve samples. Endogenous peroxidase activity was quenched by incubation in 3% H_2_O_2_ for 10 min at RT, then the slides were washed in PBS and incubated in blocking solution (5% NGS, Millipore, Massachusetts, USA) for 1 h at RT. Slides were incubated with anti-CD68 antibodies (1:300 in 1% NGS) overnight at 4 °C. The day after, slides were washed in 1% NGS and then incubated with a HRP-labeled goat anti-mouse secondary antibody (1:200 in PBS, Millipore, Massachusetts, USA). The antigen–antibody complex was detected by incubating the sections with 3,3-diaminobenzidine hydrochloride (ImmPACT® DAB Substrate Kit SK 4105 Vector Laboratories, Peterborough, UK). Negative controls were incubated only with the secondary antibody. Sections were counterstained with Haematoxylin and mounted in BioMount HM (BioOptica, Milano, Italy). CD68 immunoreactive area (brown staining) was identified and measured using the “Pixel Classifier” function of the Aivia image analysis software (Leica Microsystems, Bellevue, WA). Pixel Classifier is one of Aivia’s most popular features based on machine learning algorithms. We trained the software in order to automatically recognize three classes: macrophages (CD68 immunoreactive area), the nerve fibers and the background. When the training was completed, we launched the automatic segmentation and the software was able to recognize and measure the area (m^2^) of each segmented class. We then added up the area of each identified macrophage in order to obtain the total CD68 immunoreactive area. The degree of macrophage infiltration along the whole nerve longitudinal section was quantified by calculating the ratio between CD68 immunoreactive area and total nerve fiber area expressed as percentage.

### Statistical evaluation

All assessments were made by researchers blinded to animal treatments. The results of body weight measurements, dynamic, plantar and cold tests, IENF density, IHC, qPCR and Simoa assay were statistically analyzed using a nonparametric Mann–Whitney test (significance level set at *p* < 0.05). Morphometric data were analyzed using an unpaired t-test (significance level set at *p* < 0.05). GraphPad Prism 8 statistical package (GraphPad Software, San Diego, CA, US) was used.

## Results

### PTX and OHP induce sensory abnormalities

The body weight of the treated animals did not decrease over time during PTX and OHP treatments (Supplementary Fig. 1). Although significant differences between chemotherapy-treated animals and respective vehicles were reported, especially for animals treated with OHP (Supplementary Fig. 1b), no animal exhibited evident signs of distress nor was affected by a body weight decrease > 20% from the beginning of the study, and no signs of general or injection site intolerance were noted.

**Fig. 1:**
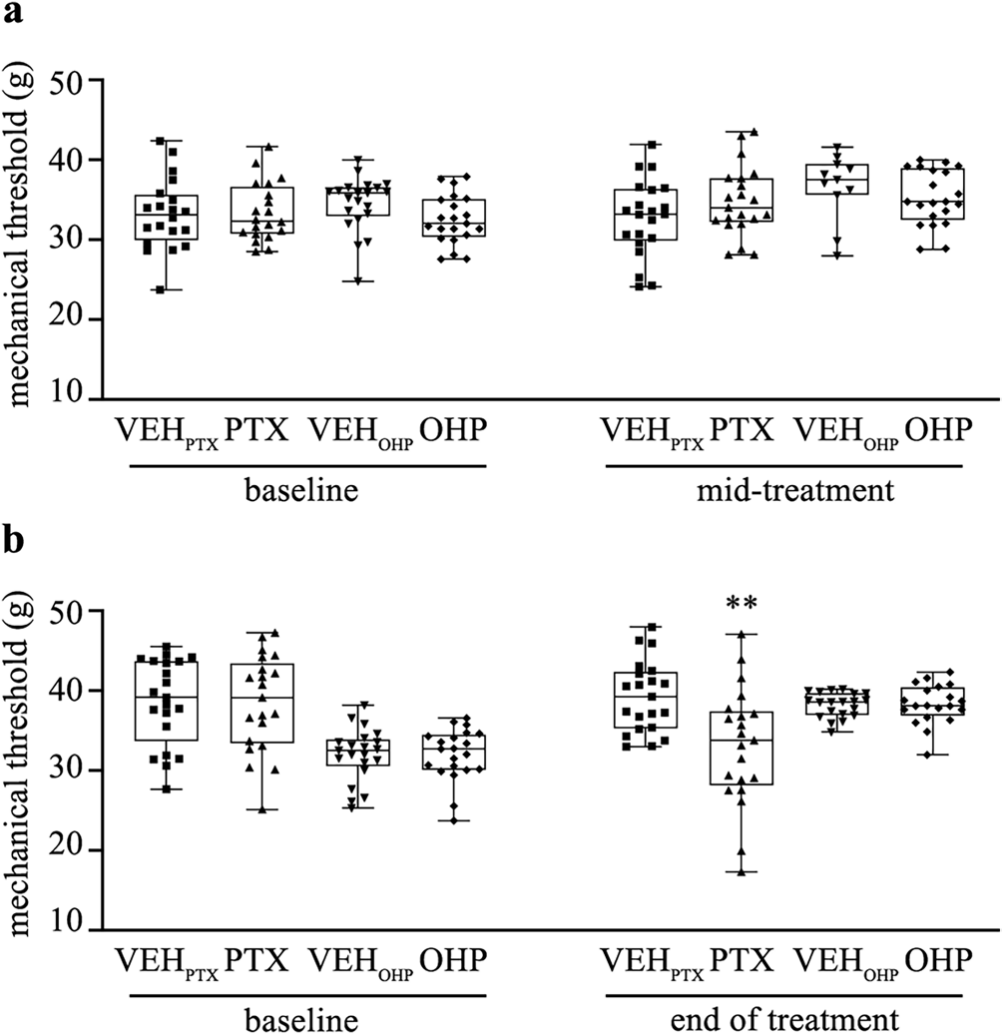
Effect of PTX and OHP on mechanical withdrawal threshold in CIPN models. (**a**) Box plots showing the mechanical thresholds of animals treated with vehicle, PTX, or OHP at baseline and mid-treatment (**b**) Box plots showing the mechanical thresholds of animals treated with vehicle, PTX, or OHP at baseline and at the end of treatment. Only PTX induced significant mechanical allodynia at the end of treatment. **p < 0.01 as compared to VEH_PTX_, Mann–Whitney test. Data are expressed as median and quartile values as well as maximum and minimum values. n = 21 for each experimental group VEH_PTX_ / VEH_OHP_, vehicle of PTX or OHP; PTX, paclitaxel; OHP, oxaliplatin.

Here, we selected specific behavioral tests in accordance with the symptomatic alterations shown by the patients treated with PTX and OHP. The assessment of the mechanical withdrawal threshold of rats exposed to PTX or OHP is presented in Fig. 1, where no significant differences among groups was recorded at baseline evaluations, while a marked impairment in mechanical threshold indicating allodynia was recorded only in PTX-treated animals at the end of treatment (*p* < 0.01) (Fig. 1b). In addition, plantar test was specifically carried out for PTX-treated animals given its noted effects on thermal thresholds, while for OHP-treated rats we performed the cold plate test, given its known cold hypersensitivity. In detail, animals treated with PTX developed signs of thermal abnormalities at the end of treatment, in which we observed an increased sensitivity to heat thermal stimulation (thermal hyperalgesia, *p* < 0.01), whereas animals treated with OHP, showed a cold hyperalgesia at the end of treatment, detected as an increase of the sign number of pain behaviors in OHP-treated animals respect to vehicle (*p* < 0.05) (as represented in Table 1). Collectively, these results suggest the development of specific sensory abnormalities in PTX- and OHP-treated animals consistent with clinical practice.

**Table1:** Effect of PTX and OHP on thermal hyperalgesia and cold hyperalgesia in CIPN models. The heat thermal thresholds and cold sensitivity (as measures of the development of sensory abnormalities) were measured by plantar test and cold plate test, respectively. Data are expressed as a mean ± SEM. *p < 0.05 as compared to VEH_OHP_ **p < 0.01 as compared to VEH_PTX_, using a nonparametric Mann–Whitney test. VEH_PTX_ / VEH_OHP_, vehicle of PTX or OHP; PTX, paclitaxel; OHP, oxaliplatin.

Plantar test (SEC—MEAN ± SEM)			Cold plate test (N SIGNS IN 300 SEC—MEAN ± SEM)		
2 weeks of treatment			3 weeks of treatment		
	baseline	2 weeks mid-treatment		baseline	3 weeks mid-treatment
VEH_PTX_	12.94 ± 0.58	12.78 ± 0.60	VEH_OHP_	14.81 ± 1.55	13 ± 1.25
PTX	13.33 ± 0.44	13.60 ± 0.58	OHP	15.10 ± 1.58	13.76 ± 1.18
4 weeks of treatment			6 weeks of treatment		
	baseline	4 weeks end of treatment		baseline	6 weeks end of treatment
VEH_PTX_	13.27 ± 0.41	14.25 ± 0.61	VEH_OHP_	20.90 ± 2.13	13.52 ± 1.11
PTX	12.87 ± 0.46	11.95 ± 0.58 **	OHP	18.70 ± 2.66	25.26 ± 4.56 *

### PTX and OHP induce peripheral neuropathy of different severity

**Fig. 2:**
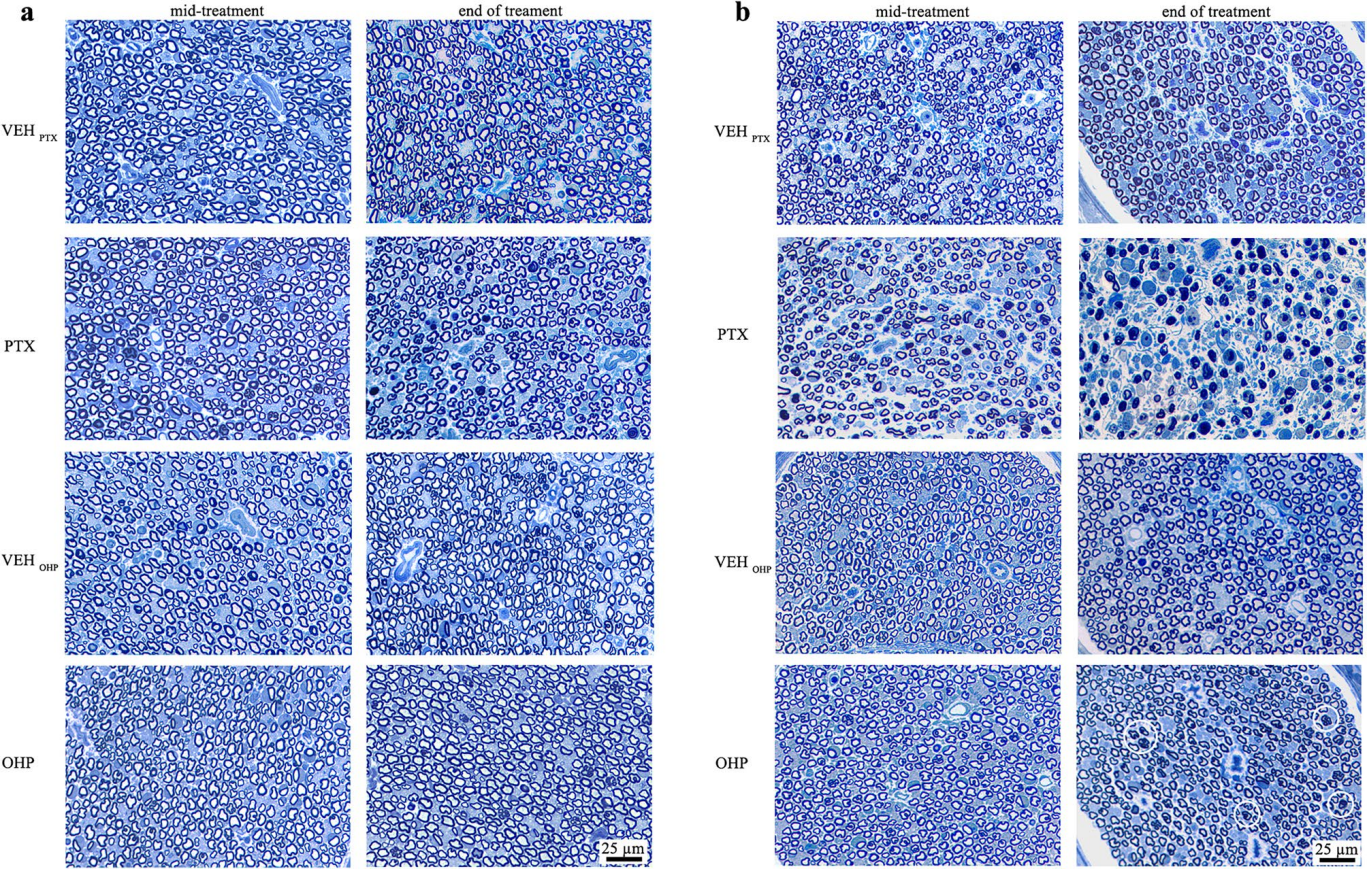
Histopathology of proximal and distal caudal nerves collected after PTX and OHP treatment. Representative images of proximal (**a**) and distal (**b**) caudal nerves of OHP- , PTX- and VEH–treated rats. Several myelinated fibers presented degeneration at different stages of severity after treatment with PTX and OHP treatments (white circles). VEH_PTX_ / VEH_OHP_, vehicle of PTX or OHP; PTX, paclitaxel; OHP, oxaliplatin.

To evaluate PTX and OHP toxicity, histopathological analysis was performed in proximal and distal caudal nerve sections of different groups at mid-treatment or at the end of treatment. Treatment with PTX induces only mild morphological changes in the proximal caudal nerve (Fig. 2a), whereas the distal portion is severely affected by PTX, with marked loss of all types of fibers and severe degeneration of myelinated fibers in comparison with OHP- and VEH-treated groups (Fig. 2b). This degeneration has been confirmed by morphometrical analysis indicating a significant loss of myelinated fibers already at mid-treatment (11,973 ± 1,329 fibers/mm^2^ in PTX versus 17,509 ± 247 fibers/mm^2^ in VEH-treated), while the very low presence of measurable fibers did not allow a quantitative measurement of the damage at the end of treatment. Moreover, pathological features compatible with axonopathy were reported after 6 weeks of OHP treatment at the level of distal caudal nerves compared with proximal portions and VEH animals.

#### OHP-treated rats induce significant decrease of neuronal soma, nucleus and nucleolus size in DRG

**Fig. 3:**
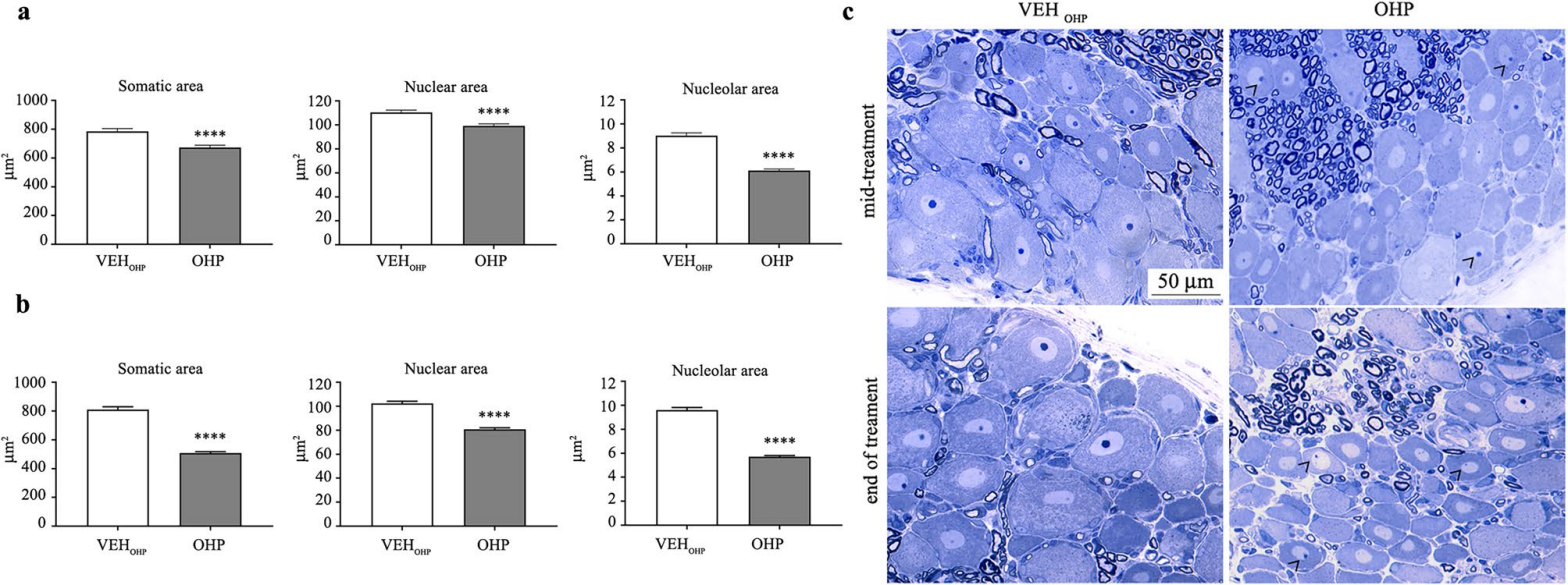
Effect of OHP on DRG sensitive neurons. At mid (**a**) and at the end of treatment (**b**), OHP was able to significantly reduce the soma, nucleus and nucleolus area of DRG sensory neurons. Representative images in (**c**) show examples of smaller DRG neurons with eccentric nucleoli (black arrowheads). ****p < 0.0001 as compared to VEH_PTX_, unpaired t-test analysis. Data are expressed as mean ± SEM. VEH_PTX_ / VEH_OHP_, vehicle of PTX or OHP; PTX, paclitaxel; OHP, oxaliplatin.

To further characterize OHP neurotoxicity, we evaluated the alteration of soma, nucleus, and nucleolus area at the level of DRG sensory neurons at mid and the end of treatments with OHP. As shown in Fig. 3, OHP treatment induced a significant decrease in neuronal soma, nucleus, and nucleolus size (*p* < 0.001) at both time points, which is a typical feature of OHP-induced neurotoxicity. Moreover, OHP induced typical morphological alterations in DRG sensory neurons at the light microscope, as previously reported^20,25,26^ characterized by small neurons and eccentric nucleoli in treated animals compared to VEH (Fig. 3c).

#### PTX and OHP treatments induce decrease of IENF density

**Fig. 4:**
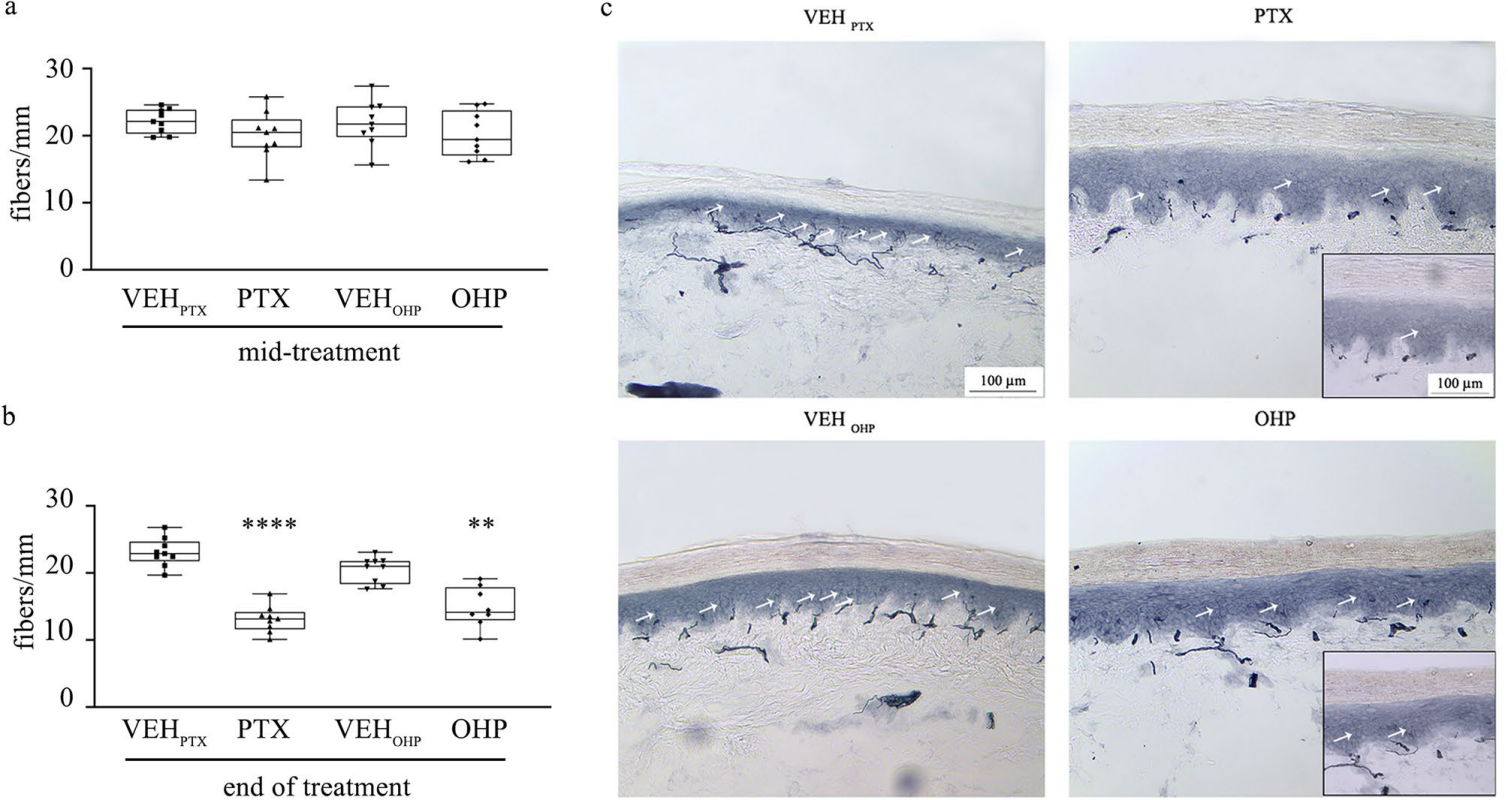
Effect of PTX and OHP on intraepidermal fibers density (IENF). Box plots showing IENF density at mid (**a**) and at the end of treatment (**b**). Only at the end of treatment PTX and OHP were able to induce a significant reduction in IENF density. Representative images in (**c**) show the reduced density of intraepidermal fibers at the end of treatment. Insets in (**c**) show higher magnification. ****p < 0.0001 as compared to VEH_PTX_, **p < 0.01 as compared to VEH_OHP_, Mann–Whitney test. Data are expressed as median and quartile values as well as maximum and minimum values. VEH_PTX_ / VEH_OHP_, vehicle of PTX or OHP; PTX, paclitaxel; OHP, oxaliplatin.

As the density of unmyelinated fibers in the skin is modified in several conditions where neuropathic pain is prominent such as after chemotherapy, we quantified PTX and OHP treatment effect at this level in both mid and end of treatment (Fig. 4a and b, respectively). IENF density was significantly reduced only at the end of treatment in both PTX- (*p* < 0.0001) and OHP-treated (*p* < 0.01) rats compared with respective VEH-treated animals (Fig. 4b). Representative images of samples collected at the end of treatment confirmed a severe decrease density of intraepidermal fibers in both chemotherapy-treated animals versus respective VEH-treated ones (Fig. 4c).

#### PTX but not OHP treatment induces macrophage infiltrates in caudal nerves

**Fig. 5:**
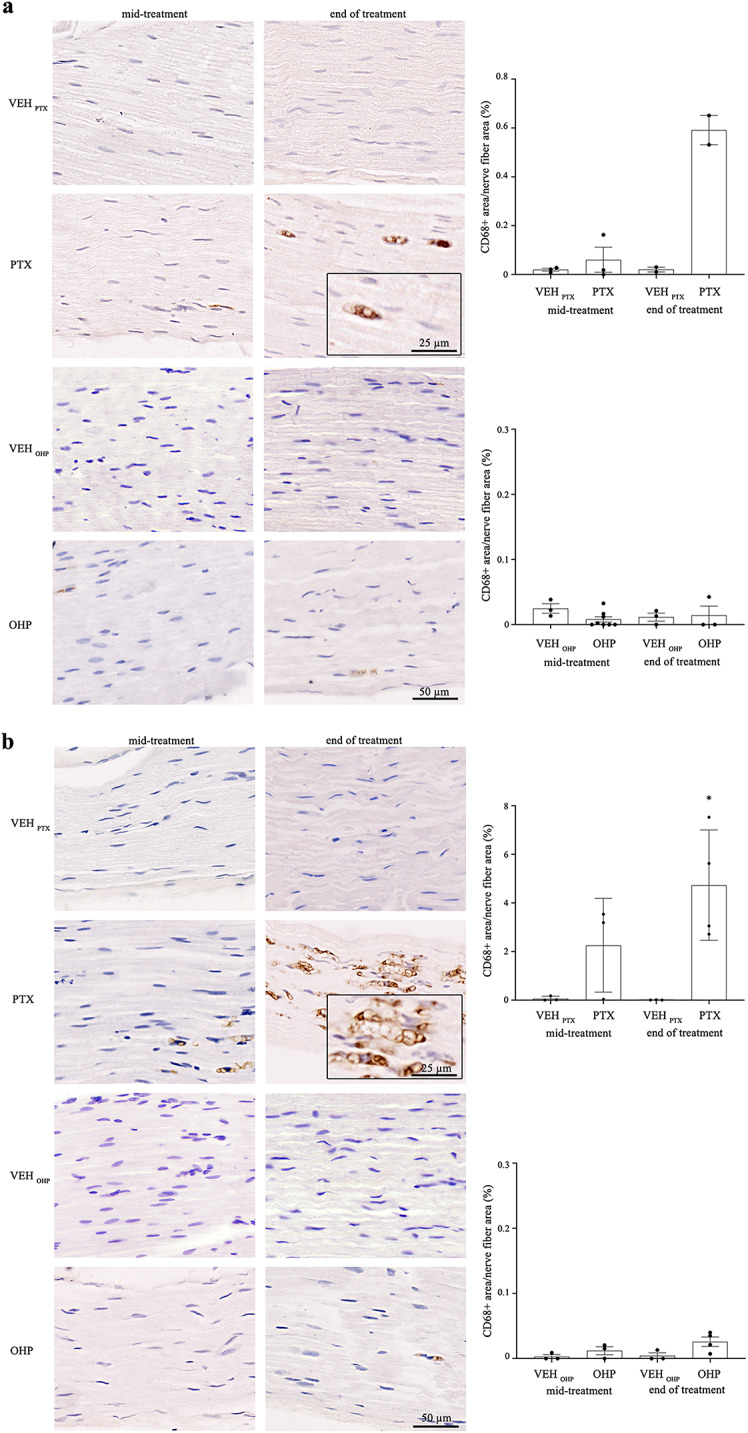
Evaluation of CD68 + macrophages infiltration in the caudal nerve of PTX- and OHP-treated rats. Representative images of CD68 + macrophages (brown staining) in the proximal (**a**) and distal parts (**b**) of caudal nerves in VEH and PTX-treated or OHP-treated animals at mid-treatment and at the end of treatment. Insets in (**a**) and (**b**) show higher magnification. Histograms show the quantification of macrophage infiltration performed by calculating the ratio between CD68 + area and total fiber nerve area expressed in percentage. *p < 0.05 as compared to VEH_PTX,_ using a nonparametric Mann–Whitney test. Values are mean ± SEM. VEH_PTX_ / VEH_OHP_, vehicle of PTX or OHP; PTX, paclitaxel; OHP, oxaliplatin.

Immunohistological analysis of CD68 marker showed a significant increase of macrophage infiltration in the distal part of the caudal nerves at the end of PTX treatment (p < 0.05, Fig. 5b), although a trend increased of macrophage infiltration at the level of the proximal caudal nerve was already present halfway through treatment (Fig. 5a). In particular, the distal parts of the caudal nerve showed a much higher density of CD68 + cells compared to the proximal portion. On the contrary, OHP treatment did not induce any significant increase in macrophage infiltration at any time.

#### PTX treatment induces IL-6 and GRO/KC (CXCL1) release in the rat serum

**Fig. 6:**
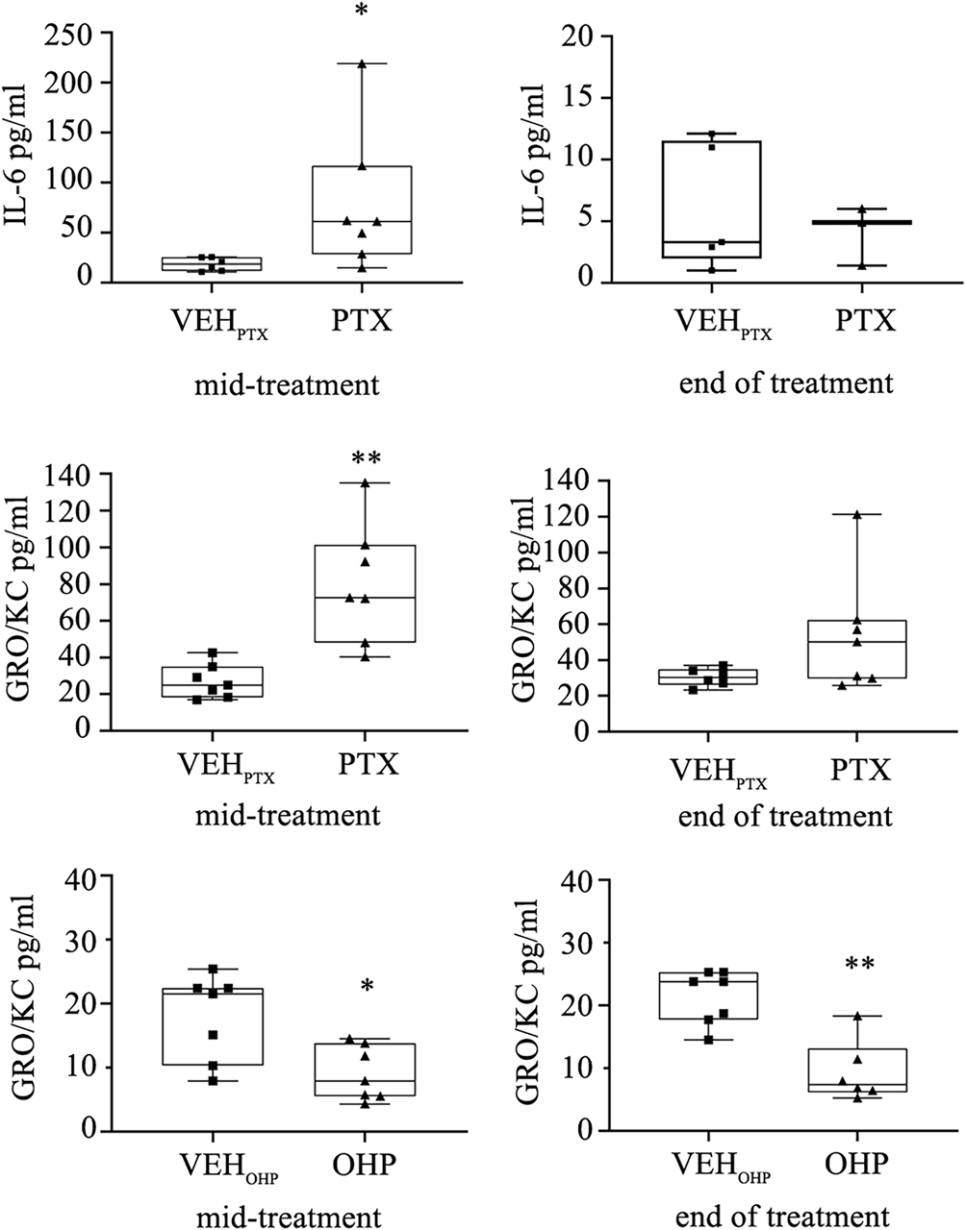
Effect of antineoplastic drugs on inflammatory cytokines levels evaluated in serum of rat treated with PTX. Box plots showing the fold change in levels of selected cytokines after treatment with PTX and OHP. After 2 weeks a significant increase of IL-6 and GRO/KC(CXCL1) levels were observed in the serum of PTX-treated rats. Data are expressed as median and quartile values as well as maximum and minimum values. n = 7 for each experimental group *p < 0.05 or **p < 0.01 as compared to VEH_PTX_, using a nonparametric Mann–Whitney test. VEH_PTX_ / VEH_OHP_, vehicle of PTX or OHP; PTX, paclitaxel; OHP, oxaliplatin.

To test the proinflammatory changes systematically, serum samples were obtained from rats at mid-treatment, and at the end of PTX or OHP administration. Here we measured the level not only of IL-6, but also of other inflammatory mediators, such as TNFα, IL-2, IL-1β, IFNγ, GRO (growth-related oncogene)/KC (also called CXCL1, a member of the CXC family of chemokines) in VEH and treated animals (Fig. 6). In the IL-6 VEH mid-treatment group one sample was under quantification limit, IL-6 VEH end-treatment group had 4 samples under detection limit, IL-6 PTX end-treatment group had 6 samples under detection limit, while for GRO/KC detection, no samples were under detection limit.

At mid-treatment, only the cytokine IL-6 and CXCL1 were significantly elevated in PTX-treated (and not in OHP-treated) rats compared to VEH (*p* < 0.05 and *p* < 0.01, respectively). At the end of treatment, both serum CXCL1 and IL-6 levels returned close to the VEH levels (Fig. 6). The levels of all the other cytokines TNFα, IL-1β, IL-2, IL-10, IFN-γ included in the panel analyzed were undetectable in both VEH and drug-treated rats. Although some animals showed outlier results, the variability remained within expected ranges, and all tested animals were included in the analysis.

#### PTX treatment induces increase of proinflammatory cytokines expression in spinal cord, caudal nerve and DRG

**Fig. 7:**
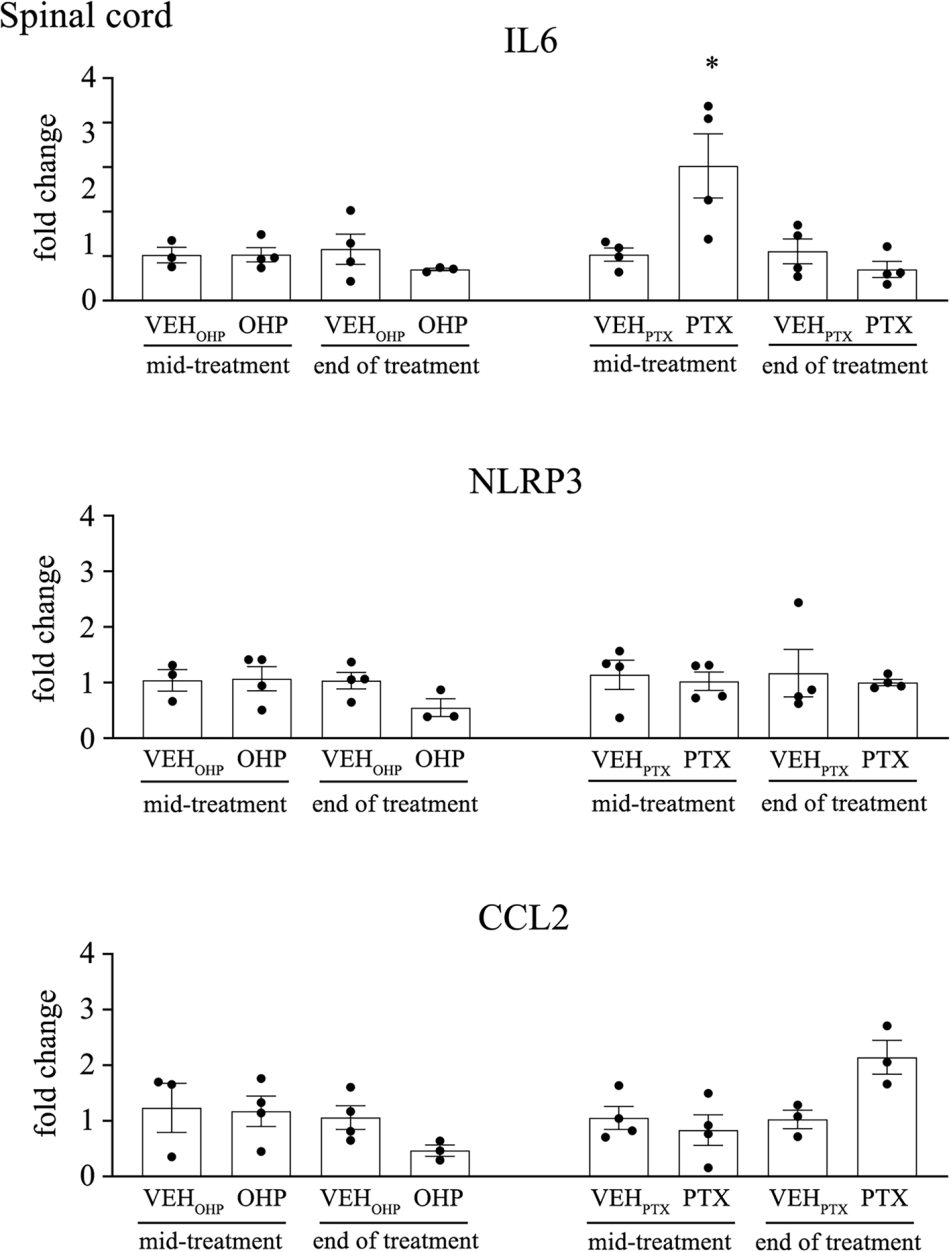
Expression of central proinflammatory cytokines in spinal cord after PTX and OHP treatments. Bar graphs showing fold change in expression of NLRP3, IL-6, CCL2 in spinal cord at mid-treatment and end of treatment. For each analyzed protein, the values were compared to that of the corresponding vehicle (VEH). Data are expressed as a mean ± SEM. *p < 0.05 as compared to VEH_PTX_, using a nonparametric Mann–Whitney test. VEH_PTX_ / VEH_OHP_, vehicle of PTX or OHP; PTX, paclitaxel; OHP, oxaliplatin.

**Fig. 8:**
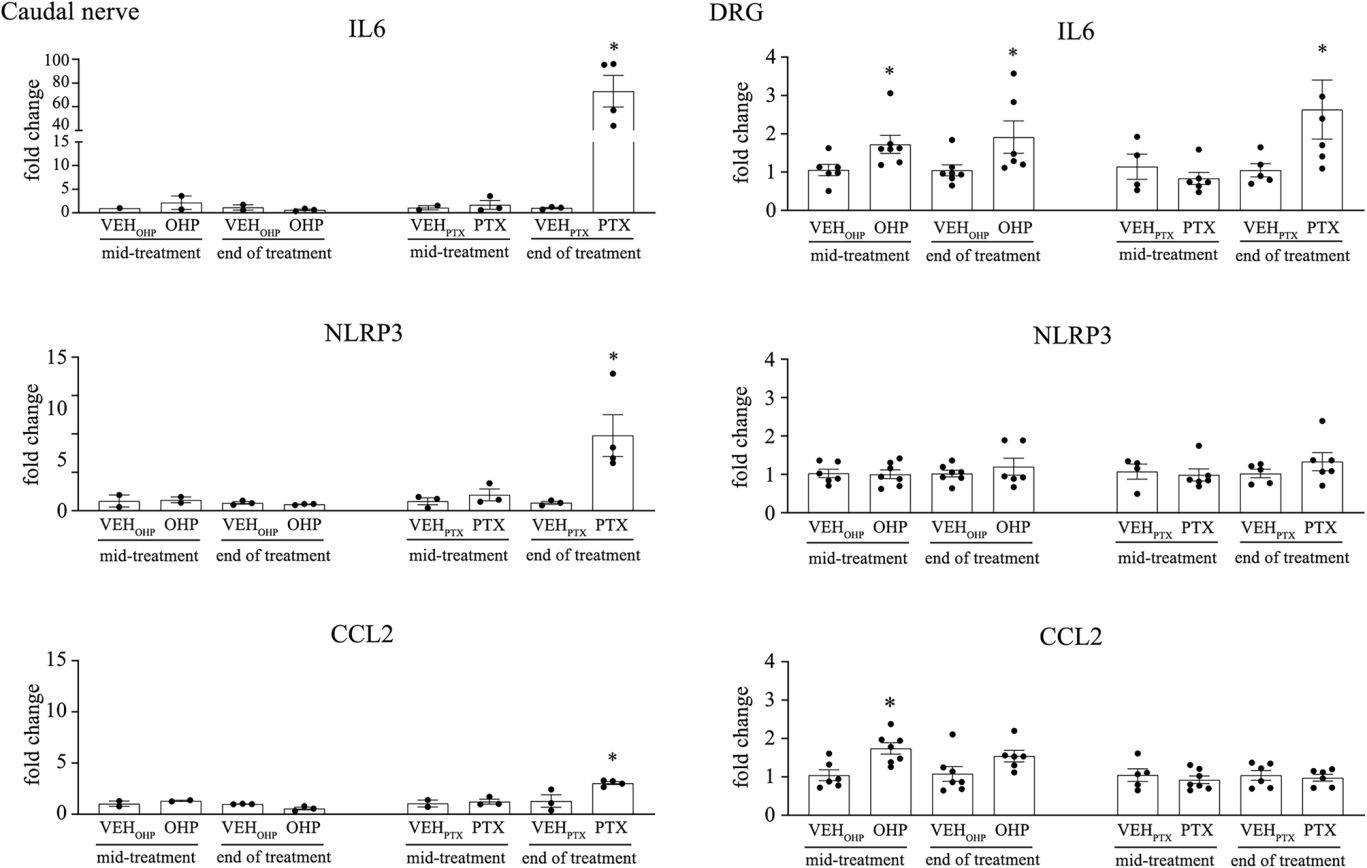
Expression of peripheral proinflammatory cytokines in caudal nerve and DRG after PTX and OHP treatments. Bar graphs showing fold change in expression of NLRP3, IL-6, CCL2 in caudal nerve and DRG at mid-treatment and end of treatment. For each analyzed protein, the values were compared to that of the corresponding vehicle (VEH). Data are expressed as a mean ± SEM. *p < 0.05 as compared to VEH_PTX_, using a nonparametric Mann–Whitney test. VEH_PTX_ / VEH_OHP_, vehicle of PTX or OHP; PTX, paclitaxel; OHP, oxaliplatin.

To identify the inflammatory processes present in tissue of nervous system, expression of proinflammatory cytokines IL-6, CCL2 and inflammasome proteins NLRP3 were analyzed in spinal cord, caudal nerve and DRG. mRNA expression of IL-6 was significantly increased in the spinal cord in PTX-treated animals at mid-treatment (*p* < 0.05, Fig. 7), and in the DRG at the end of treatment (*p* < 0.05, Fig. 8). Moreover, the expressions of IL-6 (*p* < 0.05), NLRP3 (*p* < 0.05), and CCL2 (*p* < 0.05) were significantly increased in the caudal nerve in PTX-treated animals at the end of the treatment (Fig. 8). For OHP-treated animals we were able to see significant increases of IL-6 and CCL2 at mid-treatment (*p* < 0.05), and IL-6 (*p* < 0.05) at the end of treatment in DRG (Fig. 8).

## Discussion

Since neuroinflammation has been implicated in several neuropathic pain models and its role in CIPN pathology is becoming increasingly evident, our goal was to compare two models of CIPN caused by PTX and OHP inducing remarkable different nervous tissue changes to identify serum and tissue neuroinflammation components.

Chemotherapy treatment, in addition to its impact on dividing cells, can modulate various elements of the immune system, including changes in cytokine/chemokines expression and intracellular pathways in immune cells. This modulation can contribute to the development of neuroinflammation and damage of the sensory neurons of the peripheral nervous system^10,27–29^. The activation of the inflammatory cascade, the upregulation of proinflammatory cytokines, and the involvement of neuro-immune communication pathways are suggested as factors in the initiation and progression of CIPN. Damage to the nerve, associated with Schwann cell disruption, triggers the migration of macrophages, leading to the subsequent release of cytokines and chemokines^30–32^.

In our models, we confirmed the onset of peripheral neurotoxicity in both CIPN models via morphological and morphometrical analysis. PTX, in fact, was able to induce severe fiber degeneration in peripheral nerves characterized by significant neuropathological alterations, mechanical allodynia, heat thermal hyperalgesia, and loss of intraepidermal small unmyelinated fibers, while OHP damaged firstly and mostly dorsal root ganglia (DRG) sensory neurons, reducing neuronal somatic, nuclear and nucleolar size, with a milder effect on the peripheral nerves and cold hyperalgesia. Despite severe distal nerve destruction induced by PTX treatment, no animal exhibited evident signs of distress nor was it affected by a severe body weight reduction; this is totally aligned with our previously reported data using the same model^22,33^ and are not surprising or in contrast with the available literature, since the general toxicity of the treatment is minimal. Animals’ outwardly normal behavior aligns with the limited severity of behavioral changes. Nevertheless, the severe pathological alterations were confined distally to the caudal nerve, a location where such damage is not expected to produce motor deficits detectable by standard health monitoring. In fact, the model we are using has been designed in order to achieve the full spectrum of PTX-induced peripheral neurotoxicity evidence, while other models (where behavioral changes are more severe) are focused only on neuropathic pain induced by PTX, but without any pathological evidence of peripheral nerves axonal loss: this different approach can explain the apparent discrepancy. Regarding other PTX-induced CIPN models where behavioral changes are even more prominent, an extensive review of the available data^34^ already showed that the comparison between different experimental settings in terms of sex, age, species could show different results, highlighting the need for a careful selection of the most appropriate model depending on the aims of the study. Consequently, a comprehensive model needs to demonstrate not only behavioral aspects but also the underlying nerve pathology observed in patients.

Focusing on OHP-induced peripheral neuropathy, although the clear mechanisms of neurotoxicity are not yet known, several studies in the literature confirm the reduction of the soma, nucleus and nucleolus following chronic treatment with OHP^20,25,26^. Probably, the shrinking of these organelles after OHP treatment is a strong indicator of cellular distress and represents a neuron under metabolic and structural impairments.

This morphological “atrophy” could be a manifestation of the underlying molecular damage due to platinum accumulation in the DRG neurons, which are vulnerable due to the absence of a robust blood–brain barrier. This causes a critical loss of the neuron’s functionality, causing the debilitating symptoms of OHP-induced neuropathy and often leading to irreversible nerve fiber loss.

On the contrary, probably due to a primary action of PTX on axonal mitochondria or microtubule^31^, PTX does not induce any of these changes in DRG sensory neurons, as previously reported^21^. As an additional and relevant difference, we showed an increased infiltration of macrophages in the distal part of the caudal nerve at the end of treatment with PTX administration, with a beginning appearance of macrophages already after two weeks of treatments. This finding suggests a progressive and region-specific immune response, with early macrophage activation occurring distally and a more pronounced infiltration proximally over time. Such findings indicate that macrophage recruitment may contribute to both the initiation and maintenance phases of PTX-induced nerve damage. Instead, OHP-treated rats did not show any relevant macrophage infiltrate in caudal nerves at any time. This data demonstrates that the two patterns of CIPN progression are driven by distinct initial mechanisms. It is described that the infiltration of macrophages triggers the subsequent production and secretion of various cytokines (TNFα, IL-1β, IL-6, IL-8), chemokines (CCL2, and those of the CXC family), growth factors, and inflammatory mediators generating feedforward positive loops. In addition, Schwann cells undergo phenotype modulation upon release of TNFα, IL-1β, IL-6, PGE2, ATP, leukemia inhibitory factor (LIF), and CCL2^27,35^.

These molecules are recognized as potential contributors to the onset of peripheral neuropathy by fostering the infiltration of macrophages and triggering neuroinflammation^30^.

Consistent with previous reports in the literature, in the caudal nerves of PTX-treated rats, we observed a significant increase of proinflammatory cytokines/chemokines such as IL-6 and CCL2 and the NLRP3 which is responsible for IL-1β secretion. IL-6 showed the most relevant increase in mRNA expression in the caudal nerve at the end of the treatment, while OHP-treated animals showed in DRG an increase of IL-6 and CCL2 at the mid-treatment, and an increase of IL-6 at the end of the treatment. However, since it has been known that OHP accumulates in DRG^36^, expression of proinflammatory cytokines in DRG after OHP treatment was an expected result. Although multiple studies suggest that OHP treatment generally does not induce macrophage infiltration in the DRG^37–39^, few evidence have reported macrophage infiltration when associated with neuropathic pain^40,41^.

Therefore, as different studies showed an increase of satellite glial cells and its activation in DRG after OHP^22,38,42,43^ and PTX^22^ treatment, it is possible that satellite cells contribute to the increase of cytokines that we observed in our study due to antineoplastic toxic effects. In addition, IL-6 is known to be associated with central microglia activation and plays a role in chronic pain^44^, as reported in our results after two weeks of PTX-administration.

In our model, IL-6 increase was noted in serum at mid-treatment in PTX-treated rats and subsequently presence in the peripheral nerves tissue expression could be correlated to maintenance of neuropathic pain^45–49^. This temporal sequence suggests that systemic IL-6 elevation may act as an early signal of neuroinflammatory activation preceding its local accumulation in nerve tissue. Moreover, the sustained expression of IL-6 within peripheral nerves highlights its potential role as a key mediator linking systemic inflammation to persistent nociceptive sensitization. The role of IL-6 in pain symptoms was further shown as well for post-chemotherapy women with breast cancer. Patients experiencing CIPN, in fact, exhibited notably higher levels of IL-6 and its soluble receptor sIL-6R compared to those without CIPN symptoms. These findings suggest that IL-6 trans-signaling could be the crucial biological mechanism linked to the persistence of painful symptoms^50,51^ in PTX-induced neuropathy. Moreover, circulating IL-6 as well was correlated to the axonal damage. Interestingly, painful diabetic polyneuropathy (DPN) patients that showed elevated serum levels of IL-6 represent its correlation with indicators of sensory and motor axonal damage in large nerve fibers^49^. Moreover, IL-6 has been suggested as a contributing factor in the progression of DPN^52^ and it was described that patients experiencing painful DPN exhibited higher levels of serum IL-6 than those with painless DPN^52^. Different studies in rodent neuropathic pain model showed that blocking IL-6 improves electrophysiological parameters of animals^53^ and reduces allodynia^54–57^. Interestingly, IL-6–/– mice treated with PTX did not develop typical behavioral, electrophysiological or histological signs of neuropathy at all^53^ . Temporal dynamics of IL-6 expression in serum and tissue, revealed in our study, provide valuable insights into the optimal timing for IL-6 blockade to effectively prevent disease progression.

Noteworthy, in serum collected from our PTX-treated animal, we observed an increase of chemokine CXCL1 after 2 weeks of PTX treatment. CXCL1 exerts its effects, particularly in neutrophil chemotaxis, by interacting with CXCR1 and CXCR2 receptors^58,59^. CXCL1 is primarily recognized for its involvement in the early stages of inflammation^60^. It could play direct roles within the nervous system, potentially influencing pathological pain. Neurons and various cells in the central nervous system express both CXCL1 and its main receptor CXCR2, under both normal and pathological conditions^61–63^. In vitro studies suggest that paclitaxel can induce CXCL1-associated neurotoxicity^64^. Moreover, it is proposed that CXCL1 is involved in neuropathic pain by activating CXCR2 in spinal microglia, leading to inflammatory responses and the activation of non-neuronal cells. These mechanisms may initiate peripheral and central sensitization, alter sensory pathways and induce a state of hyperexcitability through the upregulation of receptors and inflammatory mediators in the DRG and spinal cord. Among these processes, glial cells within the spinal cord, particularly microglia and astrocytes, are crucial for releasing proinflammatory mediators like cytokines and chemokines, contributing to the onset of neuropathic pain. An increase in CXCR2 expression was noted in dorsal horn neurons in the spinal and DRG neurons in various neuropathic pain models, including spinal nerve ligation (SNL)^65^ and neuropathy induced by chemotherapeutic agents^66^. Under normal conditions, microglia do not express CXCR2. However, under specific pathological conditions, these cells are activated, leading to the expression of CXCR2. It is possible that in neuropathic pain, CXCL1 could be released by spinal astrocytes, activating the heightened CXCR2 expression in spinal microglia, thereby contributing to central sensitization^67^. Interestingly, it is suggested that CXCL1 may also play a significant pro-nociceptive role through direct interactions with sensory neurons. Given that the majority of small diameter DRG neurons function as nociceptors, the notable increase in excitability observed after CXCL1 incubation, including the ability to continuously fire during prolonged depolarizations, is likely to significantly enhance sensitivity to painful stimuli in vivo. This effect occurs both by enhancing the sensitivity of nociceptors to stimuli and by increasing the release of excitatory neurotransmitters in the dorsal horn. Pro-inflammatory chemokine CXCL1, which exhibits a marked increase in DRG during the early phase of various pathological pain models, may exert significant pro-nociceptive effects, partly through its direct actions on nociceptive neurons^68^. Wang and colleagues reported that the heightened increase in the chemokine CXCL1 is associated with alterations in Na + channel expression in small diameter sensory neurons, leading to enhanced excitability and spontaneous activity in models of pathological pain induced by nerve injury or inflammation. Although CXCL1 has been noted to play a role in neuroinflammation, the complete picture of its mechanism is not clear. Moreover, up to now there have been no studies in the literature that evaluate change of CXCL1 in serum and our results, that showed IL-6 and CXCL1 increase in serum after PTX chemotherapy treatment is important to deepen its evaluation as an indicator of neuroinflammatory process in CIPN.

This deep analysis of the time course of neuroinflammatory cytokines revealed an important role for CXCL1 and IL-6 in driving neuroinflammation. However, we could not precisely discern the relative time course of the two factors: the lack of an intermediate time point leaves us without a precise temporal progression of the cytokine’s expression. Moreover, our study revealed a correlation between neuroinflammation and IL-6 and GRO/KC expression levels, but we cannot infer anything about possible causal effects of the cytokines investigated and neuroinflammatory symptoms after PTX treatment. Investigating more in depth the functional role of these cytokines in PTX-dependent neuroinflammation in our animal models using specific blockers or time-inducible knockout animals would be an interesting development of this work and add important information about precise timing of intervention to prevent CIPN and neuroinflammation in oncological patients.

## Conclusions

Although both drugs are significantly correlated with neuroinflammation, we were able to identify a rapid mobilization of IL-6 and CXCL1 in serum along with an inflammatory process only in PTX-induced neurotoxicity which leads to home immune cells such as macrophages and potentially neutrophiles to the area of injury. Moreover, since IL-6 was detected in DRG treated with OHP, this cytokine might be an important player for neuroinflammation in both CIPN models, although with different timing and sites of inflammation. These observations underline the heterogeneity of immune responses elicited by different chemotherapeutic agents and point toward drug-specific pathogenic pathways in CIPN. Importantly, our findings highlight that systemic inflammatory signatures, such as IL-6 and CXCL1, may distinguish PTX-induced neuropathy from OHP-induced neuropathy, providing a potential avenue for biomarker-guided monitoring. Moreover, the differential immune activation observed suggests that anti-inflammatory or immune-modulating strategies might be more effective in PTX-induced CIPN, while alternative protective approaches may be required for OHP-related neuropathy.

## Supplementary Information

Supplementary Information.

## Data Availability

The raw data of the study are archived and available at 10.17632/pfb2cwpt5x.1.
