# Supplementary material for: Interleukin-6 as a keystone cytokine in experimental rat models of chemotherapy-induced peripheral neurotoxicity
Source: Sci Rep. 2026 Jan 13;16:5127. doi: 10.1038/s41598-025-34830-6 (PMC12876870; doi:10.1038/s41598-025-34830-6)
Supplement: Supplementary file 1 — Supplementary Information. [file 41598_2025_34830_MOESM1_ESM.pdf]

Supplementary Information for the manuscript

INTERLEUKIN-6 AS A KEYSTONE CYTOKINE IN EXPERIMENTAL RAT MODELS OF CHEMOTHERAPY-INDUCED PERIPHERAL NEUROTOXICITY

Olga Tarasiuk[1,2], Alessia Chiorazzi[1,2], Alberto Argentini [1], Elisa Ballarini [1,2], Annalisa Canta[1,2], Valentina Alda Carozzi[1,2], Eleonora Pozzi[1,2], Paola Alberti[1,2,3], Valentina Fabbro[1], Federico Iseppon[1], Maria Foti[1], and Cristina Meregalli *[1,2]

1. School of Medicine and Surgery, University of Milano-Bicocca, Monza, Italy
2. NeuroMI (Milan Center for Neuroscience), University of Milano-Bicocca, Monza, Italy
3. Fondazione IRCCS San Gerardo dei Tintori, Monza, Italy

* Corresponding author:
Cristina Meregalli
School of Medicine and Surgery, University of Milano-Bicocca, via Cadore 48, 20900 Monza (MB), Italy. Tel: +39 02 6448 8142; e-mail: cristina.meregalli@unimib.it; ORCID: 0000-0002-4281-4577

Supplementary Figures

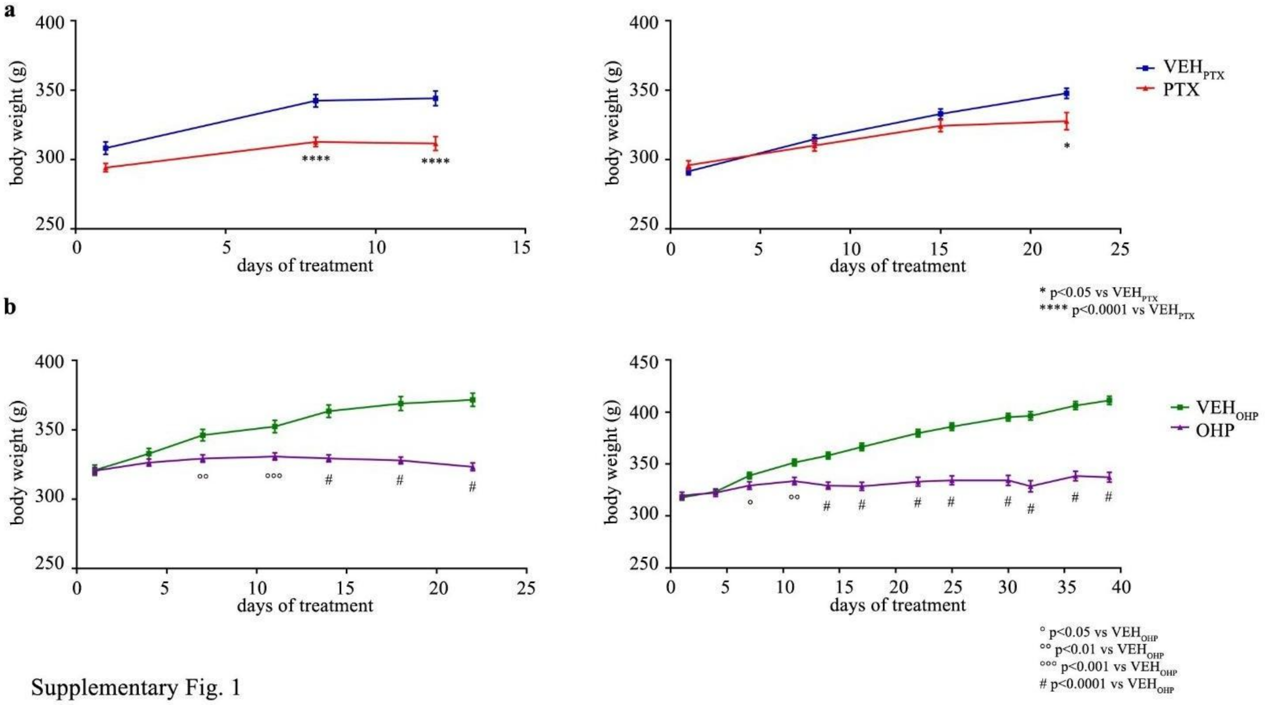

Supplementary Fig. 1. Effect of PTX and OHP on body weight in CIPN models. (a) Plots showing the body weight course over time of animals treated with vehicle or PTX for two (left) and four weeks (right). (b) Plots showing the body weight course over time of animals treated with vehicle or OHP for three (left) or six weeks (right).

n=21 for each experimental group. VEH_{PTXPTX} / VEH_{OHPOHP} vehicle of PTX or OHP; PTX paclitaxel; OHP oxaliplatin. Data are expressed as mean ±SEM. *$p < 0.05$; ****$p<0.0001$ as compared to VEH_{PTX}.; °$p < 0.05$; °°$p<0.01$; °°°$p<0.001$; #$p<0.0001$ as compaed to VEH_{OHP} using a nonparametric Mann-Whitney test.
